# The Role of GSK-3 in Cancer Immunotherapy: GSK-3 Inhibitors as a New Frontier in Cancer Treatment

**DOI:** 10.3390/cells9061427

**Published:** 2020-06-09

**Authors:** Giuseppa Augello, Maria R. Emma, Antonella Cusimano, Antonina Azzolina, Giuseppe Montalto, James A. McCubrey, Melchiorre Cervello

**Affiliations:** 1 Institute for Biomedical Research and Innovation, National Research Council (CNR), 90144 Palermo, Italy; giuseppa.augello@irib.cnr.it (G.A.); mariarita.emma@irib.cnr.it (M.R.E.); antonella.cusimano@irib.cnr.it (A.C.); antonina.azzolina@irib.cnr.it (A.A.); giuseppe.montalto@unipa.it (G.M.); 2 Department of Health Promotion, Mother and Child Care, Internal Medicine and Medical Specialties, University of Palermo, 90127 Palermo, Italy; 3 Department of Microbiology and Immunology, Brody School of Medicine at East Carolina University, Greenville, NC 27834, USA; MCCUBREYJ@ecu.edu

**Keywords:** GSK-3, immunotherapy, NK cells, T cells, PD-1, small molecule inhibitors

## Abstract

The serine/threonine kinase glycogen synthase kinase-3 (GSK-3) was initially identified because of its key role in the regulation of glycogen synthesis. However, it is now well-established that GSK-3 performs critical functions in many cellular processes, such as apoptosis, tumor growth, cell invasion, and metastasis. Aberrant GSK-3 activity has been associated with many human diseases, including cancer, highlighting its potential therapeutic relevance as a target for anticancer therapy. Recently, newly emerging data have demonstrated the pivotal role of GSK-3 in the anticancer immune response. In the last few years, many GSK-3 inhibitors have been developed, and some are currently being tested in clinical trials. This review will discuss preclinical and initial clinical results with GSK-3β inhibitors, highlighting the potential importance of this target in cancer immunotherapy. As described in this review, GSK-3 inhibitors have been shown to have antitumor activity in a wide range of human cancer cells, and they may also contribute to promoting a more efficacious immune response against tumor target cells, thus showing a double therapeutic advantage.

## 1. Introduction

Glycogen synthase kinase-3 (GSK-3) is a ubiquitously expressed serine/threonine kinase. It is a multifunctional monomeric protein involved in various cellular functions, including differentiation [1], survival [2], glycogen metabolism [3,4], protein synthesis [5], immune responses [6], and cell death [7]. It is a central component of many pathways, including those mediated by the Insulin/Insulin receptor (IR), Insulin-like growth factor (IGF)/IGF receptor (IGFR), Wnt/β-catenin [8], Hedgehog [9], nuclear factor κB (NF-κB) [10], and Transforming Growth Factor-β (TGF-β) signaling pathways [11]. In mammals, there are two isoforms of GSK-3 encoded by separate genes, named GSK-3α (51 kDa) and GSK-3β (47 kDa). The two isoforms differ significantly in their N-terminal regions, yet have similar catalytic domains. The most studied form of GSK-3 is GSK-3β, which has two main domains: a β-strand domain present at the N-terminus, and an α-helical domain present at the C-terminus, where kinase activity is located. An ATP-binding site is present at the interface of the two domains. GSK-3β activity is regulated by phosphorylation at two different sites. Phosphorylation of the Serine 9 (Ser9) site inactivates GSK-3β, whereas phosphorylation at the Tyrosine 216 (Tyr216) site increases its catalytic activity. Many upstream kinases are known to phosphorylate the Ser9 of GSK-3β, including protein kinase A (PKA) [12], AKT/PKB [13], PKC [14], p90 ribosomal S6 kinase/MAPK-activating protein (p90RSK/MAPKAP), and p70 ribosomal S6 kinase (p70S6K) [15]. GSK-3β activation, regulated by Tyr216 phosphorylation, is constitutively active in resting cells. This phosphorylation site is regulated by other protein tyrosine kinases, such as SRC-family tyrosine kinases, Fyn [16], and MEK1 [17].

To date, about 40 proteins involved in a large number of cellular processes have been definitively validated as GSK-3β substrates, and over five hundred proteins have been proposed as GSK-3β substrates [18]. GSK-3β can regulate gene expression by activating or inhibiting some transcription factors, such as β-catenin, NF-κB, the nuclear factor of activated T cells (NFAT) [19], cyclic AMP response element binding protein (CREB) [20], c-Jun, and AP-1 [21].

GSK-3β is a pivotal element in the phosphatidyl-inositide 3-kinase (PI3-Kinase) and Wnt signaling pathways. GSK-3β was one of the first substrates of the oncogenic kinase AKT to be identified. Growth factors and anti-apoptotic stimuli activate PI3-Kinase/PDK1, thus leading to AKT kinase phosphorylation which, in turn, phosphorylates GSK-3β at Ser9, resulting in its inactivation. Moreover, AKT/GSK-3β signaling also controls levels of β-catenin. Indeed, GSK-3β acts as a negative regulator of Wnt/β-catenin, taking part in a multiprotein destruction complex. 

Furthermore, GSK-3β has been widely shown to regulate inflammation through its role in controlling the stability of two important transcription factors involved in inflammatory response, i.e., NF-κB and CREB. The active form of GSK-3β can phosphorylate NF-κB and CREB, promoting their proteasomal degradation. On the contrary, GSK-3β inhibition induces the migration of NF-κB and CREB into the nucleus and regulates the production of different pro- and anti-inflammatory cytokines [6]. 

Despite evidence of a substantial anti-survival role of GSK-3β in some cancers, GSK-3β may promote the growth and survival of cancer cells through several mechanisms. It has been shown that active GSK-3β phosphorylates and stabilizes NF-κB essential modifier (NEMO) [22], leading to its interaction with IκB kinase (IKK). Activated IKK phosphorylates the NF-κB inhibiting IκBs, promoting their degradation via the proteasome pathway and thus leading the free NF-κB to translocate to the nucleus where it promotes gene transcription. Therefore, GSK-3β inhibition may repress the activity of NF-κB, resulting in the downregulation of various genes involved in cancer progression, such those coding for anti-apoptotic proteins.

Active GSK-3β can inhibit the apoptotic pathway through the phosphorylation of MDM2, a GSK-3β target and a negative regulator of p53. Notably, the inhibition of GSK-3β blocks the activation of MDM2 and induces apoptosis by stabilizing p53 [23]. Moreover, it has been reported that in mutant KRas-dependent human tumors, GSK-3 has pro-survival activity, whereas β-catenin and c-Myc have pro-apoptotic activity [24]. Importantly, in this scenario, GSK-3 inhibition prevented the phosphorylation of its substrates c-Myc and β-catenin, and consequently promoted their upregulation, which led to c-Myc- and β-catenin-dependent tumor suppression [24]. Furthermore, a pro-survival role of GSK-3β has been reported through the modulation of Notch signaling. The Notch signaling pathway promotes cancer proliferation, invasion, and resistance to therapy [25,26]. Some studies have shown that active GSK-3 phosphorylates Notch, a GSK-3 target, preventing its degradation via the proteasome pathway, and as a result it is stabilized, promoting its enhanced nuclear localization and transcriptional activity [27]. 

The dysregulation of GSK-3β has been implicated in various human diseases, such as diabetes [28], cancer [29,30], bipolar mood disorder [31], liver diseases [29,32], and neurodegenerative diseases [33]. The modulation of GSK-3β activity, with the use of small molecule compounds capable of blocking or activating GSK-3β, may form a valuable strategy to control such diseases. Indeed, lithium chloride (LiCl) was the first GSK-3 inhibitor to be approved by the US FDA for the treatment of bipolar disorder.

This review has been constructed to highlight the potential therapeutic relevance of GSK-3 as a target for anticancer therapy, pointing out the value of GSK-3 as a target in cancer cells as well as a target in immune cells, i.e., NK cells and T cells, since newly emerging data have demonstrated its pivotal role in regulating immune cell functions. For these purposes, preclinical (in vitro and in vivo animal model experiments) and clinical studies were searched using relevant keywords in various databases, such as PubMed, ScienceDirect, SpringerLink, Scopus, Google Scholar, and ClinicalTrial.gov, as well as abstracts from international meetings (up to April 2020).

## 2. GSK-3 Inhibitors

Many GSK-3 inhibitors have been described, various of them have been tested in preclinical studies (Table 1), and some are under evaluation in clinical studies (Table 2). Many of these inhibitors are of synthetic origin, whereas others have natural origins [29]. Moreover, they can be divided into three main categories: non-ATP-competitive inhibitors, ATP-competitive inhibitors, and substrate-competitive inhibitors [29]. 

An important factor in cell growth signaling and apoptosis control, GSK-3β is a potential therapeutic target in cancer. Some GSK-3 inhibitors have been used in animal tumor models, either alone or in combination with chemotherapeutic drugs. Tideglusib, a non-ATP-competitive GSK-3β inhibitor, has been tested in xenograft and PDX murine models of human glioblastoma [34,35].

AR-A014418, an ATP-competitive and selective GSK-3β inhibitor, has been reported to suppress the proliferation and induce the apoptosis of gastric cancer [36], synovial sarcoma, and fibrosarcoma cells in vitro as well as in vivo [37].

TWS119 is a specific inhibitor of GSK-3β which has been shown to inhibit cell proliferation and induce the apoptosis of human alveolar rhabdomyosarcoma cells [38], while it regulates epithelial-mesenchymal transition (EMT) and cancer stem cell (CSC) properties in triple-negative breast (TNB) cancer [39].

LY2090314 is an ATP-competitive GSK-3 inhibitor which inhibits both GSK α and β isoforms. Recently, in an in vitro study, Kunnimalaiyaan et al. demonstrated for the first time, in a neuroblastoma (NB) model, that LY2090314 inhibited the growth of both human MYCN amplified and non-amplified NB cells [40]. LY2090314 has also been evaluated in vivo using an orthotopic xenograft nude mouse model of pancreatic cancer [41]. LY2090314 given in combination with nab-paclitaxel (Abraxane^®^) significantly prolonged the median survival of mice. However, LY2090314 and nab-paclitaxel as single agents were completely ineffective.

9-ING-41 is an ATP-competitive inhibitor of both GSK-3α and GSK-β isoforms that has been reported to have significant anticancer activity in in vitro and in vivo studies. It has been tested both as a single agent and in combination with standard cytotoxic chemotherapies and novel target-specific agents in a range of solid tumors, such as neuroblastoma [42], pancreatic [43], glioblastoma [35], and bladder [44] cancers, as well as in hematological malignancies, such as lymphoma [45,46].

Two GSK-3 inhibitors, LY2090314 and 9-ING-41, have been evaluated, or are under investigation, in clinical trials for cancer treatment (Table 2). LY2090314 has been evaluated in two completed clinical trials of patients with advanced or metastatic cancer, in combination with pemetrexed and carboplatin (NCT01287520) [47], as well as in patients with acute leukemia (NCT01214603) [48], while another study was terminated due to the low number of patients enrolled (NCT01632306). In the two completed studies, LY2090314 was shown to be safe and well tolerated, and was associated with antitumor activities when used in combination with other agents [47]; however, it showed limited clinical benefit as a single agent [48].

The Actuate 1801 phase 1/2 study (NCT03678883) is recruiting patients to evaluate the safety and clinical efficacy of 9-ING-4, both as a single agent and in combination with chemotherapeutic drugs, in patients with refractory solid tumors or hematologic malignancies. Nevertheless, to date, no GSK-3β inhibitor has been approved for treatment of these complex diseases.

Emerging data on GSK-3 as a regulator of components of the immune response also suggest the possibility of using GSK-3 as an immunotherapeutic target. Hence, GSK-3 inhibitors, at least in some tumor types, can act on cancer cells per se and, as described below, they can also act on cells of the immune system, thus showing a double therapeutic advantage.

## 3. Background of Immune System Cells and Immune Checkpoint Proteins

The human immune system consists of two principal acting arms: innate and adaptative immune response. In general, innate immune response is useful for creating a first cellular barrier against infective pathogens or malignant transformed cells, and for creating an inflammatory micro-environment with the production of several cytokines that favor the activation of cells involved in adaptative immune response. Immune checkpoint proteins are regulators of key processes in the immune system. In physiological conditions, these molecules are the modulators of the signaling pathways responsible for immunological tolerance, preventing the destruction of “self” cells in the organism by the immune system. These proteins are expressed on the cell surface of different cell types of the innate and adaptative immune systems, such as Natural Killer (NK) cells and T cells [49,50].

NK cells are the main actors of innate immune response, the first cellular barrier against viral or microbial infections. Unlike T lymphocytes, NK cells are unable to recognize pathogens, but they are specialized in destroying and neutralizing virally infected or malignant transformed cells through a mechanism called “missing-self” [51,52]. 

NK cells express a wide spectrum of transmembrane receptors on their surface that have stimulatory or inhibitory effects on the activation and modulation of immunological response [53,54]. In general, NK inhibitory receptors, such as Killer Immunoglobulin Receptors (KIRs), Leucocyte Immunoglobulin Receptors (LIRs), CD96, Lymphocyte Activation Gene 3 (LAG-3), are characterized by an extracellular domain which recognizes Human Leucocyte Antigen (HLA) molecules expressed on target cells, and from 1 to 3 cytoplasmic domains with the immunoreceptor tyrosine-based inhibition motif (ITIM). Upon binding with their specific ligands on the extracellular domain, the phosphorylation of specific tyrosine residues on ITIM domains induces the activation of inhibitory receptor-mediated signaling. 

The association of inhibitory receptors with HLA molecules expressed on target cells plays a key role during NK cell development and education for self-recognition [55], preventing immune response against healthy host cells and counteracting tissue damage during the activation of immune response against microbes or viral infections. 

The co-inhibitory activity of KIRs and LIRs can be opposed by the expression of co-activating NK receptors such as NKp30, NKp46, NKp44, and NKG2D that recognize ligands selectively expressed in virally infected cells or in malignant transformed cells [56]. However, the activation of NK cells, and therefore cytotoxicity toward target cells, depends on the co-engagement of specific activating receptors, such as NKG2D and 2B4, or NKG2D and DNAX Accessory Molecule-1 (DNAM-1), which do not activate on their own. 

**Figure 1:**
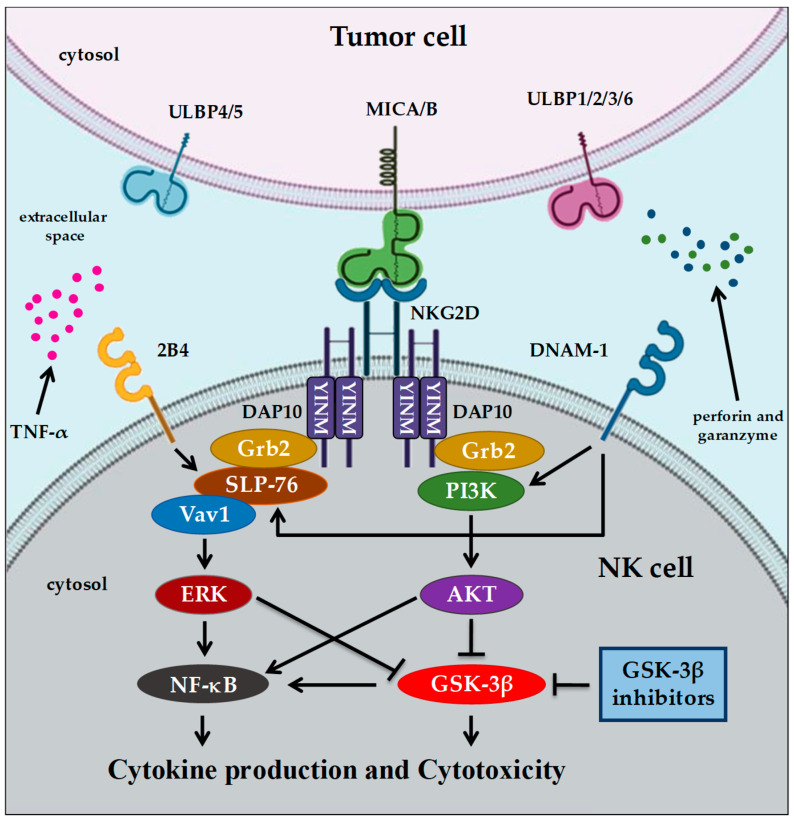
A simplified overview of NK cell activation by the NKG2D pathway. NKG2D upon binding to its specific ligands, MICA/B or ULBPs, associates with adaptor protein DAP10, which is responsible for transducing intracellular activating signaling. NK cell activation depends on the co-engagement of specific activating receptors, such as NKG2D and 2B4, or NKG2D and DNAM-1. These co-engagements induce activation of SLP-76, resulting in Vav1-dependent activation of ERK. Once activated, ERK activates NF-κB or inhibits GSK-3β. NKG2D or DNAM-1 activates another signaling cascade through PI3K and AKT, resulting in GSK-3β inhibition and NF-κB activation. Activation of NF-κB leads to cytokine production and cytolysis of target cells by granzymes and perforins.

NKG2D is a homodimeric transmembrane receptor which, upon binding with its specific ligands, the major histocompatibility complex (MHC) class I chain-related protein A (MICA)/B [57] and the HCMV glycoprotein UL16-binding protein family molecules (ULBPs) [58], associates with its adaptor, DAP10, which is responsible for transducing intracellular activating signaling [59]. The DAP10 cytosolic tail, in fact, contains a YINM domain which induces the recruitment of PI3K and the Grb-Vav complex and, consequently, activates the AKT and ERK pathways leading to cytokine production and the cytolysis of target cells by granzymes and perforins [58] (Figure 1). 

T cells are the main effectors of adaptative immune response. Unlike the innate arm of the immune system, adaptative immune response may act specifically against pathogenic antigens that are recognized as “not-self” by T cells, through a mechanism known as antigen presentation. The activation of “naïve” T cells requires interaction between T Cell Receptors (TCRs) and antigenic ligands exposed on MHC-I or MHC-II molecules expressed by antigen presenting cells (APCs). 

Evidence demonstrates that TCR/antigen/MHC-I/II interaction is not sufficient to induce complete T cell activation [60], which instead requires additional co-stimulatory signals to become fully efficient, inducing the production of specific cytokines and T cell proliferation. CD28 is the main co-stimulatory receptor expressed on the T cell membrane which may interact with its specific ligands, CD80 and CD86, expressed on APCs. It has been reported that, without CD28 co-stimulation, both CD4+ and CD8+ T cells undergo a hyporesponsive status called anergy [61]. The anergic state of T cells can be considered a mechanism of immunological tolerance that prevents the development of self-reactive T cells [62]. Many authors have reported mechanisms for rescuing anergic T cells involving the PI3K/AKT signaling pathway, the modulation of sirtuin 1 activity, and cytosolic Ca2+ mobilization [63,64]. The co-stimulating activity of CD28 receptor can be inhibited by the expression of Cytotoxic T-Lymphocyte Antigen 4 (CTLA-4), also known as CD152, on the T cell membrane.

CTLA-4 is a glycoprotein receptor, expressed on CD4+, CD8+, and regulatory T cells (Treg) cells, that may act as an immune checkpoint, suppressing immune response during the initial activation of naïve T cells. CTLA-4 recognizes the same ligands as CD28 (i.e., CD80 and CD86); however, while CD28 is constitutively expressed on the T cell membrane, the expression of CTLA-4 may occur only after TCR stimulation [65]. Since CTLA-4 binds the CD80 and CD86 ligands with higher affinity than CD28, even low expression levels of CTLA-4 can compete with CD28 for ligand binding, switching off the activation of the immune response. 

**Figure 2:**
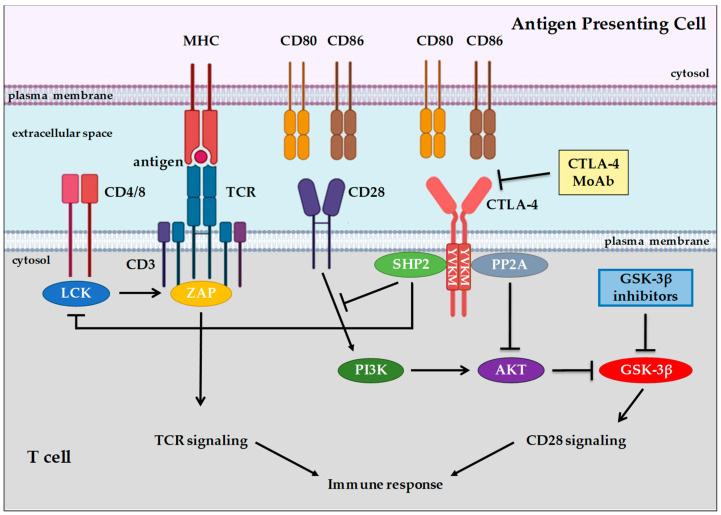
A simplified overview of the TCR and CD28 signaling pathways in T cells. CTLA-4 competes with CD28 for ligand binding, switching off immune response activation. Upon binding to its specific ligands, the homodimerization of CTLA-4 induces SHP2 dependent dephosphorylation of specific TCR proximal proteins, such as CD3, suppressing the activation of immune response. CTLA-4 binding induces association with PP2A, resulting in CTLA-4-dependent AKT signaling pathway inhibition. CTLA-4 binding may also induce association with phosphatase SHP2, which dephosphorylates CD28, thus preventing recruitment of PI3K, activation of AKT, and inhibition of GSK-3β, leading to a reduction in T cell activation.

At the molecular level, CTLA-4 has a structure similar to CD28 with an extracellular V domain, a transmembrane domain, and a cytoplasmic tail without intrinsic catalytic activity, containing one YVKM motif able to bind PI3K, PP2A, and SHP2, and one proline-rich motif able to bind SH3 containing proteins: upon binding to its specific ligands, the homodimerization of CTLA-4 induces PP2A/SHP2 dependent dephosphorylation of specific TCR proximal proteins, such as CD3, suppressing the activation of immune response [66] (Figure 2). CTLA-4 binding induces the activation of PI3K/AKT signaling that, in turn, inactivates GSK-3β, achieving T cell anergic state [66]. Therefore, CTLA-4 is constitutively expressed on Treg and mediates their suppressive functions on effector T cells [67]. 

Another important immune checkpoint that acts during the effector phase of T cell activation is programmed death receptor 1 (PD-1). PD-1 is a glycoprotein monomeric receptor structurally similar to CTLA-4 and CD28: it is characterized by an extracellular immunoglobulin-like domain, a transmembrane domain, and a cytoplasmic tail containing an ITIM and an immunoreceptor tyrosine-based switch motif (ITSM) [68]. Unlike CTLA-4, whose expression is exclusive to activated T cells, PD-1 expression is also found in B cells and myeloid cells [69,70]. In addition, whereas CTLA-4 ligands are expressed exclusively by professional APCs, the expression of PD-1 ligands, known as PD-L1 and PD-L2, characterizes a wide range of cellular types, including tumor cells. PD-L1 is expressed by T cells, B cells, and can be expressed in parenchymal cells upon interferon γ (IFN-γ) stimulation or the activation of tumorigenic signaling pathways [70]. PD-L2 is mainly expressed by professional APCs [71], but its expression can be induced in a great variety of immune and non-immune cells in response to different micro-environmental stimuli [72,73,74,75]. 

**Figure 3:**
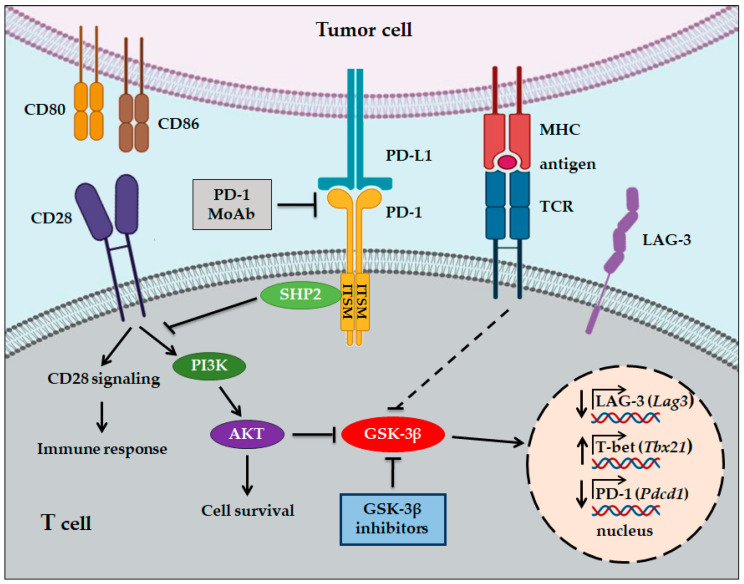
A simplified overview of the CD28, PD-1, and TCR signaling pathways in T cells. Upon the binding of PD-1 with its specific ligand, SHP2 is recruited to specific sites of the ITSM motif of PD-1’s cytoplasmic tail. SHP2 dephosphorylates CD28, thus preventing recruitment of PI3K and activation of the AKT signaling pathway, leading to a reduction in T cell proliferation and activation. In the presence of anti-PD-1 MoAbs, SHP2 is sequestered by PD-1, thus allowing CD28 phosphorylation and recruitment of PI3K, which leads to GSK-3β inhibition via AKT signaling. Inhibition of GSK-3β promotes the transcription of *Lag3* (LAG-3) and *Tbx21* (T-bet). T-bet expression inhibits transcription of *Pdcd1* (PD-1). TCR-specific stimulation leads to the inactivation of GSK-3β.

Upon the binding of PD-1 with its specific ligand, the Src homology 2 domain-containing protein tyrosine phosphatase 1 (SHP1), or SHP2, is recruited to specific sites of the ITSM motif of PD-1’s cytoplasmic tail and inhibits PI3K/AKT signalisation, leading to a consistent reduction in T cell proliferation and activation [76] (Figure 3). Since PD-L1 and PD-L2 are expressed on peripheral cells, the interaction of these ligands with their receptor contributes to maintaining immunological tolerance in locally infiltrated tissues, preventing autoimmunity events [77]. In addition to CTLA-4, PD-1 expression is also found in Treg cells, even though the significance of this expression is not fully understood. 

Escaping from immunological surveillance and immune suppression are some of the strategies that cancer cells exploit to promote tumor growth and metastasis. Tumor cells can evade immunological surveillance and progress through different mechanisms, such as the activation of immune checkpoint pathways that promote the suppression of antitumor immune responses. For these reasons, as discussed below, immunotherapeutic approaches able to reactivate antitumor immune responses, by interrupting co-inhibitory signaling pathways and promoting immune-mediated elimination of tumor cells, are promising strategies for the treatment of various malignancies.

## 4. GSK-3 and Immunotherapy in Cancer

As described previously, immune cells of the innate and adaptive immune systems, such as NK and T cells, participate in immune response against cancer cells. Recent evidence has highlighted the role of GSK-3 in the regulation of immune response in cancer [5,78,79].

NK lymphocytes are important cells of the innate immune system which are able to recognize and destroy stressed cells, such as virally infected or cancer cells, without antigen-specific receptor recognition. The activation of NK cells depends on the co-engagement of specific activating receptors. The engagement of NKG2D/2B4 or NKG2D/DNAM-1 leads to GSK-3β inhibition through ERK or AKT signaling, respectively. Therefore, GSK-3β activity acts as a negative regulator of multiple NK cell activating signals. Consequently, NK cell activation and function could be enhanced by the knockdown of GSK-3β or its inhibition with different pharmacological small molecule inhibitors (SMIs).

NK cells kill cancer cells after binding to them through interaction between NK receptors, such as the activating receptor NKGD2, and cancer cell ligands, such as MICA/B and ULBPs, which are HLA-related molecules. Fionda et al. have recently shown that the inhibition of GSK-3 with LiCl, SB216763, or BIO increased MICA expression at protein and mRNA levels in human multiple myeloma (MM) cell lines, as well as in tumor cells isolated from the bone marrow of MM patients, without significant effects on expression levels of MICB or the DNAM-1 ligand PVR/CD155 [80]. In addition, treatment with GSK-3 inhibitors significantly increased NK-mediated cytotoxicity of MM cells and further enhanced MICA expression when used in combination with the chemotherapeutic drugs lenalidomide or melphalan. Furthermore, combinations significantly increased NK cell-mediated tumor killing by promoting NKG2D recognition in NK cells. From a mechanistic point of view, GSK-3 inhibition correlated with the reduced expression of activated STAT3 transcription factor, which is known to be a negative regulator of MICA transcription. Thus, GSK-3 SMIs, through the regulation of MICA expression, may be novel therapeutic agents that could improve immune response in MM patients.

NK cells from patients with acute myelogenous leukemia (AML) are known to show significantly reduced cytotoxic activity against cancer cells. Parameswaran and co-authors demonstrated that NK cells from AML patients expressed high levels of GSK-3β, and this was associated with a reduced ability of NK cells to kill AML cells [81]. Interestingly, treatment with the GSK-3 inhibitors SB415286, LY-2090314, or Tideglusib, or the genetic inactivation of one or the other of the GSK-3 isoforms, enhanced the ability of NK cells to kill AML cells, also due to increased tumor necrosis factor α (TNF-α) levels. Mechanistically, GSK-3 inhibition promoted the upregulation of lymphocyte function associated antigen 1 (LFA-1) in NK cells, and of intercellular adhesion molecule-1 (ICAM-1) on AML target cells, resulting in a stable adhesion of NK cells to their target cells and thereby promoting AML-NK cell conjugates and the subsequent killing of AML cells.

Recently, a subset of NK cells expressing NKG2D receptor and high levels of CD57, a marker of cell maturation [82], with characteristics similar to classic memory T and B cells, such as viral antigen specificity, clonal-like expansion, persistent and rapid recall response, has been discovered [83,84,85]. Some studies have reported that patients with solid cancers, with higher numbers of tumor-infiltrating NK cells expressing high levels of CD57, have a better survival rate and tumor regression [82,86,87,88]. In addition, in hematological malignancies, patients with higher absolute counts of NKG2D+ CD57+ NK cells showed lower relapse rates after hematopoietic cell transplant (HCT) [89]. These NKG2D+ CD57+ cells specifically expand in response to cytomegalovirus (CMV) infection in humans and are referred to as “adaptive” NK cells. The distinctive characteristics exhibited by adaptive NK cells make them of particular importance in the search for new cancer immunotherapies.

Despite the great clinical interest in mature adaptive CD57+ NK cells, very little is known about the cellular signaling pathway(s) that lead to late stage NK cell maturation. Recently, Chichocki et al. have demonstrated that during ex vivo expansion of NK cells with IL15, the addition of the GSK-3 inhibitor CHIR99021, an ATP-competitive inhibitor of both GSK-3α and GSK-β isoforms, significantly enhanced CD57 acquisition and maturation following the upregulation of lineage-specific transcription factors, such as E-box binding homeobox 2 (ZEB2), PR/SET domain 1 (PRDM1), and T-box 21 (TBX21) [90]. Moreover, NK cells expanded in the presence of CHIR99021 produced higher amounts of TNF-α and IFN-γ in response to target cell recognition and had greater natural cytotoxicity against a range of cancer cell lines, such as pancreatic, ovarian, and lung cancer cells, compared with conventional NK cells. Furthermore, in an in vivo xenograft model of ovarian cancer, the adoptive transfer of NK cells, expanded in the presence of CHIR99021, demonstrated superior antitumor efficacy [90]. Therefore, the inhibition of GSK-3 with pharmacological agents, through the maturation of NK cells during ex vivo expansion, may constitute a novel approach for effective cancer immunotherapy.

Based on this preclinical evidence, three clinical trials are currently evaluating the safety and objective response rate of adaptive NK cells that have been previously expanded in culture with CHIR99021 and IL-15, and then infused into patients in combination with IL-2 or monoclonal antibodies (Herceptin or Erbitux) in patients with refractory or relapsed AML (NCT03081780), ovarian cancer (NCT03213964), and other solid tumors (NCT03319459) (Table 2).

Various evidence has demonstrated the important role of GSK-3 in the functioning of T cells. GSK-3 is constitutively active in resting T cells, and it has been reported to act as a negative regulator of T cell response, as its constitutive activity inhibits CD8+ T cell proliferation and IL-2 production [91]. Conversely, TCR-CD28 engagement results in an increase in the PI3K/AKT signaling pathway, which leads to the phosphorylation and inactivation of GSK-3 activity [92] [Figure 2]. 

Also of interest are the results reported by Appleman et al. that the pharmacological inactivation of GSK-3 by LiCl in human T cells substitutes for CD28, but not for CD3, a costimulatory signal for T cell proliferation [93]. Similar results were reported by Taylor and Rudd, who demonstrated that downregulation with specific small-interfering RNAs (siRNAs) or inhibition with four different GSK-3 SMIs with distinct structures, SB415286, CHIR99021, L803mts, and SB216763, can substitute for CD28 co-stimulation in the potentiation of cytotoxic CD8+ cytolytic T cells (CTL) function against lymphoma cells expressing ovalbumin peptide (OVA257–264) [94].

GSK-3 has also been implicated in the regulation of induced regulatory Treg (iTreg) activity. Xia et al. have shown that in naïve CD4+ T cells, the pharmacological inhibition of GSK-3β by specific inhibitors, such as TDZD-8 or SB216763, stimulated iTreg differentiation and increased their suppressive activity through the activation of the TGF-β/Smad3 signaling pathway [95]. Moreover, Zhang et al. have reported that GSK-3β inhibition increased the tumor cell cytotoxic capacity of CD8+ memory stem T cells in vitro against gastric cancer cells [96]. Mechanistically, this was associated with the upregulation of the expression of FasL, granzyme B, IFN-γ, and T-bet, a transcription factor which regulates the development of naïve T lymphocytes. 

Similarly, the inhibition of GSK-3β in glioblastoma (GBM)-specific IL13 chimeric antigen receptor expressing T (IL13-CAR-T) cells increased the survival and proliferation of CAR-positive T cells upon activation with IL13Rα2-expressing GBM tumor cells, or soluble target antigen. Moreover, exposure to a target antigen increased cytotoxic efficacy and tumor-killing ability in GSK-3β-inhibited IL13-CAR-T cells [97].

All together, these data highlight the pivotal role of GSK-3 in the regulation of important T cell functions and, moreover, suggest that GSK-3 inhibitors may be new potential anticancer drugs capable of enhancing T cell antitumor activity and improving treatment with CAR-T-cells.

In recent years, immune checkpoint blockade (ICB) has proven to be efficacious in immunotherapy for various types of cancer. Since FDA approval in 2011 of the first immune checkpoint inhibitor (ICI) ipilimumab, an anti-CTLA-4 monoclonal antibody (MoAb), another six ICIs have been approved for cancer therapy: three anti-PD-1 MoAbs, nivolumab, pembrolizumab, and cemiplimab, and three anti-PD-L1 MoAbs, atezolizumab, avelumab, and durvalumab [98].

However, although the ICB of negative co-receptors on T cells, such as CTLA-4 and PD-1, is an increasingly popular approach in cancer treatment, many patients do not respond to these treatments or develop resistance to them. Therefore, there is a need to improve single agent treatment or, alternatively, to develop novel approaches in cancer immunotherapy using combinations of ICIs with each other or with molecular targeted agents. These latter approaches are currently being tested in ongoing clinical trials [98,99].

In this context, Taylor et al. have demonstrated that GSK-3 is the major regulator of PD-1 expression on T cells [94]. Knockdown with siRNAs or the inhibition of GSK-3 by the SMI SB415286 in CD8+ CTL reduced the cell surface expression of PD-1 and increased their cytolytic activity against a lymphoma cell line in vitro as well as viral clearance [100]. Mechanistically, GSK-3 inhibition, through siRNA or by SMI treatment, increased *Tbx21* (encodes T-box transcription factor, T-bet) transcription which, in turn, suppressed *Pdcd1* (encodes PD-1) gene transcription in CD8+ CTL (Figure 2). Moreover, in animal studies, the injection of GSK-3 inhibitors in mice suppressed PD-1 expression and increased T-bet expression in association with enhanced CD8+ CTL function [93]. These results suggest that GSK-3 inhibition may be an alternative approach to the use of antibodies against PD-1 in cancer immunotherapy.

Interestingly, the same group demonstrated that GSK-3 inhibition with SMIs was as effective as anti-PD-1 and PD-L1 blocking antibodies in the suppression of tumor growth of melanoma and lymphoma cells in primary tumor and metastatic mouse models [101]. Moreover, confirming previous results, treatment with GSK-3 SMIs increased *Tbx21* transcription, while inhibiting *Pdcd1* transcription and PD-1 expression on the cell surface. However, the combination of anti-PD-1 and the SMI SB415286 showed the same effect as monotherapy, suggesting that these agents are pharmacologically similar. Furthermore, using a genetic approach, the authors demonstrated that CD8+ cytolytic T cells, isolated from conditional knockout GSK-3α/β^-/-^ mice, exhibited reduced PD-1 expression and suppressed melanoma tumor growth and pulmonary metastasis to the same extent as PD-1 gene deficiency, thus suggesting the direct role of GSK-3 in the regulation of CTL antitumor function.

More recently, Rudd et al. have identified LAG-3 as a novel target regulated by GSK-3 activity in T cells [102]. They demonstrated that in vitro treatment of CD4+ and CD8+ T cells with GSK-3 SMI SB415286 downregulated the expression of LAG-3, while the expression of other inhibitor receptors, such as CTLA-4, was unaffected, except for PD-1 which, in accordance with what was previously shown by the same authors, was also downregulated. Importantly, although LAG-3 blockade alone had a limited effect on suppressing melanoma cell growth, and SB415286 showed some inhibitor effects, their combination significantly enhanced CD8+ T cell cytolytic responses against lymphoma target cells in vitro. These results were confirmed in in vivo animal studies using melanoma cells. The combination of GSK-3 SMI with anti-LAG-3 therapy significantly decreased tumor growth and, in addition, further inhibited pulmonary metastasis of melanoma cells, more than either agent alone. This combination was even more effective than the combination of anti-LAG-3 and anti-PD-1. Mechanistically, GSK-3 inhibition increased *Tbx21* transcription, therefore increasing T-bet expression levels, and this in turn promoted the binding of T-bet to the LAG-3 promoter, resulting in its inhibition (Figure 3).

GSK-3 inhibitors have also been evaluated in the context of tumor treatment using CAR-T cell immunotherapy. Sengupta et al. have reported that treatment of GBM-specific IL13CAR-T cells with the GSK-3 inhibitor SB216763 resulted in reduced PD-1 expression due to T-bet upregulation, and promoted T cell survival and proliferation [103]. In animal studies, the treatment of GBM-bearing mice with GSK-3-inhibited CAR-T cells, resulting in 100% tumor elimination in tumor-re-challenge experiments. Therefore, these results open new perspectives for the development of immunotherapeutic strategies mediated by CAR-T cells, not only against GBM but also other solid tumors.

However, all these data have been obtained in mouse models, and whether this approach is also valid in human patients remains to be demonstrated by clinical trials.

## 5. Conclusions

To date, although new anti-cancer immunotherapies based on ICIs have shown encouraging clinical responses in some types of cancer, their use in many patients shows little improvement and, in some cases, even hyperprogression [104]; thus, this type of therapy needs to be optimized. This suggests the need to develop novel approaches for anti-cancer immunotherapy, also based on combination therapies, such as combining ICIs with each other or with molecular targeted agents.

Emerging data have highlighted that GSK-3 kinase represents an important anticancer target, both as a modulator of numerous cellular pathways of tumor cells responsible of tumor growth, proliferation, and metastasis, as well as a regulator of the signaling pathways responsible for the immune response of cells of the innate and adaptative systems, mainly NK and T cells. Accordingly, GSK-3 SMIs have been shown to have antitumor activity in a wide range of human cancer cells and, as described in this review, they may also contribute to promoting a more efficacious immune response against tumor target cells, thus showing a double therapeutic advantage.

Moreover, there are certainly some advantages of using GSK-3 SMIs: (i) their low cost compared to antibodies used in immunotherapy; (ii) the fact that they can be administered orally; (iii) the fact that they do not seem to have serious side effects on healthy host cells, as demonstrated by using LiCl, a GSK-3 inhibitor, which has been used for decades for the treatment of patients with bipolar disease.

However, to date, none of the GSK-3 SMIs have been approved for anticancer therapies. So far, available data have been obtained in preclinical models and must be validated in clinical studies. These studies should also clarify whether GSK-3 SMIs, either used alone or in combination with ICIs, act on tumor cells, on T cells, or on NK cells, as described above, or on the different cell types simultaneously. Eventually, these studies will determine whether GSK-3 inhibitors will be new agents to add to the armamentarium available to oncologists for cancer immunotherapy.

## Figures and Tables

**Table 1:** GSK-3 inhibitors evaluated in preclinical studies.

Inhibitor Name	Tumor Type	Ref.
**Tideglusib**	glioblastoma	[34,35]
**AR-A014418**	gastric cancer	[36]
	synovial sarcoma	
	fibrosarcoma	[37]
**TWS119**	alveolar rhabdomyosarcoma	[38]
	breast cancer	[39]
**LY2090314**	neuroblastoma	[40]
	pancreatic cancer	[41]
**9-ING-41**	glioblastoma	[35]
	neuroblastoma	[42]
	pancreatic cancer	[43]
	bladder cancer	[44]
	lymphoma	[45,46]

**Table 2:** GSK-3 inhibitors in clinical trials (as of April 2020).

Inhibitor Name	Therapeutic Application	Clinical Trials	Status
**LY2090314**	metastatic cancer	NCT01287520	Completed
	acute leukemia	NCT01214603	Completed
	pancreatic cancer	NCT01632306	Terminated
**9-ING-4**	lymphoma/pancreatic cancer	NCT03678883	Recruiting
**CHIR99021**	NK cells expanded in culture with CHIR99021 and IL-15, and then infused into patients with acute myelogenous leukemia (AML)	NCT03081780	Active, not recruiting
**CHIR99021**	NK cells expanded in culture with CHIR99021 and IL-15, and then infused into patients with ovarian cancer	NCT03213964	Recruiting
**CHIR99021**	NK cells expanded in culture with CHIR99021 and IL-15, and then infused into patients with solid tumors	NCT03319459	Active, not recruiting
